# Microstructural deformation observed by Mueller polarimetry during traction assay on myocardium samples

**DOI:** 10.1038/s41598-020-76820-w

**Published:** 2020-11-25

**Authors:** Nicole Tueni, Jérémy Vizet, Martin Genet, Angelo Pierangelo, Jean-Marc Allain

**Affiliations:** 1 LMS, CNRS, École polytechnique, Institut Polytechnique de Paris, Palaiseau, France; 2 Inria, Palaiseau, France; 3 LPICM, CNRS, École polytechnique, Institut Polytechnique de Paris, Palaiseau, France

**Keywords:** Tissues, Biological physics, Biophotonics

## Abstract

Despite recent advances, the myocardial microstructure remains imperfectly understood. In particular, bundles of cardiomyocytes have been observed but their three-dimensional organisation remains debated and the associated mechanical consequences unknown. One of the major challenges remains to perform multiscale observations of the mechanical response of the heart wall. For this purpose, in this study, a full-field Mueller polarimetric imager (MPI) was combined, for the first time, with an in-situ traction device. The full-field MPI enables to obtain a macroscopic image of the explored tissue, while providing detailed information about its structure on a microscopic scale. Specifically it exploits the polarization of the light to determine various biophysical quantities related to the tissue scattering or anisotropy properties. Combined with a mechanical traction device, the full-field MPI allows to measure the evolution of such biophysical quantities during tissue stretch. We observe separation lines on the tissue, which are associated with a fast variation of the fiber orientation, and have the size of cardiomyocyte bundles. Thus, we hypothesize that these lines are the perimysium, the collagen layer surrounding these bundles. During the mechanical traction, we observe two mechanisms simultaneously. On one hand, the azimuth shows an affine behavior, meaning the orientation changes according to the tissue deformation, and showing coherence in the tissue. On the other hand, the separation lines appear to be resistant in shear and compression but weak against traction, with a forming of gaps in the tissue.

## Introduction

Cardiac muscle has a complex, highly hierarchical organization composed of cardiomyocytes, extracellular collagenous matrix, vasculature, etc. This organization is deeply related to the macroscopic mechanical and morphological properties of the heart. The cardiac muscle or myocardium is mostly made of an assembly of cells, the cardiomyocytes, which are roughly cylindrical with a typical diameter around 10–20 $\upmu$m, and a length around 100–150 $\upmu$m^1^. Each cardiomyocyte is surrounded by a thin layer of extra-cellular matrix, the endomysium. This defines the smallest scale of the tissue. Cardiomyocytes are bundled together, creating aggregates of typically 5–10 cells. These bundles have a typical size of around 100 $\upmu$m. These aggregates are themselves surrounded by a thick extra-cellular matrix layer, the perimysium. Both endomysium and perimysium are mostly made of collagen fibers^2^. At a larger scale, these aggregates are assembled to create the heart wall which is approximately one centimeter thick.

The cardiomyocytes are contractile cells, their main axis being the direction of contraction. These local preferential directions are thus the ones of the cardiac fibers^3^. It is now well established that the fiber orientation rotates in the thickness of the heart wall^4,5^. The spatial organization of the cell aggregates remains elusive. It is often considered that these structures create sheetlets, large layers of cardiomyocytes separated by a thick film of extra-cellular matrix^6,7^. However, this is not definitively proven and other organizations, such as globular clusters of dozens of cardiomyocytes, have also been proposed^8,9^. On top of this uncertainty on the mesoscopic organization of the myocardium, the physiological role of this mesostructure is not completely understood^10,11^.

The primary reason for this lack of knowledge of the mesostructural organization of the cardiomyocytes comes from the experimental difficulties to observe it, in particular in association with their mechanical role. Different methods have been used so far to observe the microstructure. The most resolving method is probably the Scanning Electronic Microscopy (SEM), which enables to observe, for example, the cellular shape^9,12^ or the organization of the perimysium and the endomysium^9^, showing clusters of cardiomyocytes. In these images, the perimysium appears to be much thicker and denser than the endomysium^13^. However, SEM has the major drawbacks of having a limited field of view and of requiring invasive sample preparation. Conversely, the Magnetic Resonance Imaging (MRI) has been used to investigate the local orientation of sheetlets (or at least the local anisotropy of cardiomyocyte aggregates) by measuring the magnetic relaxation^14,15^. While the MRI has the capability to observe the whole heart *in vivo*, it cannot be used to observe directly its mesostructure, which can only be inferred through theoretical assumptions. Intermediate scales have been investigated through confocal microscopy^8,16,17^. Here the collagen is specifically dyed to distinguish the endomysium and the perimysium from the cardiomyocytes. Millimeter-long lateral surfaces of rectangular pieces of the heart wall have been observed with this approach. It provides specificity, high resolution and a large field of view, but requires very long scanning times, making it incompatible with mechanical assays. Confocal microscopy images show large lines of collagen on the lateral surfaces of the sample. While some lines seem dense and thin, others appear broad and sparse. Sparse layers will tend to behave like lines of weakness, strong in compression but weak under traction, whilst denser layers will behave like stiff sheets. These contrasts raise questions about the mechanical properties of the collagen walls.

These mechanical properties, which have attracted considerable attention, can be generally split between active and passive properties^18–20^. The active response is due to the contraction of the cardiomyocytes, whilst the passive response arises from the whole tissue. Our focus is set on the latter. Various mechanical assays have been performed, such as uniaxial traction^21–24^, biaxial ones^25–27^ or shear experiments^26,28^ with the ultimate aim to determine the anisotropic mechanical response of the tissue. The anisotropy arises mainly from the cardiomyocyte orientation, but some experiments point towards a role of the cardiomyocyte bundles in the response^26,28^. However, to our best knowledge, no experiment has probed the mechanical response at the scale of the cardiomyocyte bundle (although this has been done on skeletal muscle^29^). These questions require new experimental approaches, that allow to obtain mesostructural information for a large field of view and at a rate comparable to that of the mechanical loading.

To tackle these questions and access structural information at the mesoscale, a full-field Mueller Polarimetric Imager (MPI) is used, for the first time, in this study. Over the past few years, Mueller polarimetric imaging has shown great potential for exploring the microstructure of biological tissues for different types of biomedical applications^30^. The great advantage of this technique is that it can be used to observe, in few seconds, a macroscopic field of view (several square centimeters), thus providing structural information on the explored tissue with a microscopic resolution (less than 100 $\upmu$m, which is smaller than the size of the mesostructure). This technique can thus provide unique and complementary information with respect to the other imaging techniques, at a rate compatible with mechanical assays. It exploits the polarization of the light to determine various biophysical polarimetric quantities related to the tissue’s scattering or anisotropy properties. Many studies have demonstrated that this technique provides unique enhanced contrasts which can considerably improve the detection of pathological areas on a large variety of tissues^31–33^. Recently, Mueller polarimetric imaging has been used to characterize the microstructure of healthy and infarcted myocardium^34,35^.

In this study, we investigated the role of the myocardium mesoscale organization in its mechanical response, with a particular interest in the response of the perimysium—the collagen layer which surrounds cardiomyocyte bundles—as the role of this structure remains unclear. To do so, we used full-field MPI to investigate the microstructure of the myocardium under different stretch conditions. For this purpose, an original setup, combining a full-field MPI and a mechanical traction device was built in order to measure the evolution of the different polarimetric properties of myocardium during the stretching of strip samples of pig heart wall. The proposed approach enables to quantitatively investigate the structural modification of myocardial tissue on a mesoscopic scale, via the measurement of its polarimetric parameters, under different traction conditions. We observed an affine evolution of the tissue optical orientation, indicating that the mesostructure follows the local macroscopic deformation. However, the forming of gaps, perpendicular to the traction direction, indicated the presence of defect lines. These lines reflect weaknesses in the tissue, and are associated with a lower depolarization, denoting a less dense region which considerably reduces the light scattering. Our new imaging method gives a first evidence of the mechanical response of the collagen structure surrounding the sheetlet, which appears weak in response to traction, while being resistant to shear and compression.

## Results

Nine samples of dimensions approximately $30\times 10\times 2.4\,\text{ mm}^3$ were analyzed by using the new proposed approach. They were extracted from porcine left ventricle, parallel to the heart wall. One side of these samples was marked with an ink speckle to measure the local deformation via Digital Image Correlation (DIC). The samples were then fixed at both extremities of a custom-made traction device^36^ and were kept immersed in a physiological buffered saline solution (PBS) to prevent any drying of the tissue during measurements. The samples were subjected to traction, with pauses every 10% of stretch for MPI acquisition. Three types of data were collected for each sample: (1) the Mueller polarimetric matrix at every 10% of deformation, (2) the force and the displacement of the traction machine every second and (3) the local deformation every 3 s.

### Mueller polarimetric imaging

The Mueller matrix is determined through a custom-made MPI designed and constructed at LPICM. The MPI is used to measure the sample’s Mueller matrix. It consists in a 16-components real valued matrix enabling the comprehensive polarimetric characterization of the explored sample. To retrieve the main polarimetric properties of the sample, the polar decomposition proposed by Lu and Chipman^37^ is used to extract the birefringence, the depolarization and the dichroism matrices from measured Mueller Matrix. For the myocardium, the most relevant polarimetric parameters are the Depolarization ($\Delta$), and the Linear birefringence, which is characterized by the Linear Phase Retardance (*R*) and the orientation of the fast axis, also named Azimuth ($\alpha$). The parameters $\Delta , R$ and $\alpha$ are scalar and have been computed for each pixel of the image.

The parameter $\Delta$ quantifies the scattering properties of the explored sample (0 for non-depolarizing sample, 1 for a complete depolarization). Furthermore, the parameters $\alpha$ and *R* provide a complete information on the anisotropy of the explored tissue. Specifically $\alpha$ reflects the local optical orientation of the fiber structures in the tissue. The parameter *R* indicates the difference in optical phase shifts, between two polarization directions (respectively parallel and orthogonal to $\alpha$), that the polarization of the light undergoes after reflections by the tissue.

**Figure 1:**
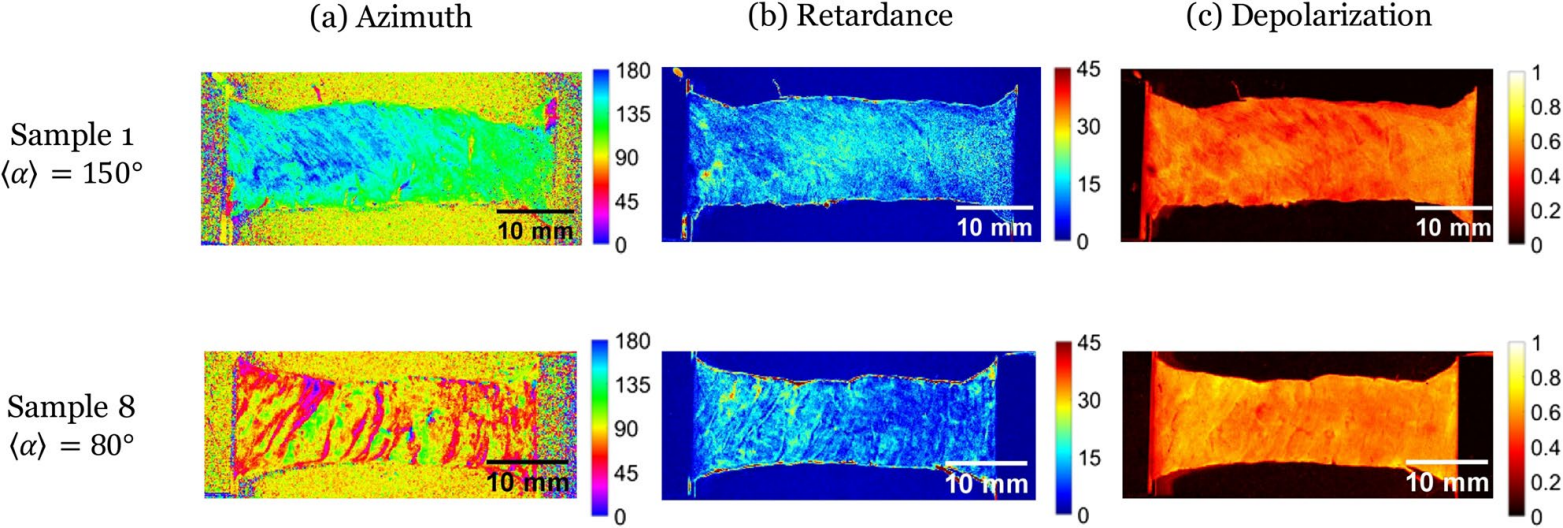
Maps of the Mueller polarimetry parameters [(**a**) Azimuth $\alpha$ (in $^{\circ }$), (**b**) retardance *R* (in $^{\circ }$), (**c**) depolarization $\Delta$] for two of our 9 samples, chosen for their different initial mean azimuth $<\alpha>$ (top—Sample 1, $\langle \alpha \rangle \approx 150^{\circ }$; bottom—sample 8, $\langle \alpha \rangle \approx 80^{\circ }$; direction of traction $0^{\circ }$).

Figure 1 shows the maps of $\alpha , R$ and $\Delta$ for two chosen samples of nine tested ones (samples numbered 1 and 8). These samples were chosen for their two different initial average value of $\alpha$ (indicated as $<\alpha>$ in the following text). Beside that they are representative of the other samples—which are all presented in supplementary data. Sample 1 has an initial value of $<\alpha > \approx 150^{\circ }$, $0^{\circ }$ being the traction direction (Fig. 1 top), whilst sample 8 presents a value of $<\alpha > \approx 80^{\circ }$ (Fig. 1 down). For both samples, the azimuth maps reveal structural patterns, with higher contrasts observed for sample 8 (with pattern perpendicular to the traction direction). This pattern is due to lines on which the parameter $\alpha$ varies brutally, indicating a sudden change in the tissue microstructure orientation. We call these lines “separation lines”. Linear retardance *R* and depolarization $\Delta$ maps are much less contrasted, even though they still show a pattern reflecting the separation lines.

Although the separation lines are observed on all samples, the contrast is stronger for samples with an average $<\alpha>$ perpendicular to the traction direction ($<\alpha >\approx 90^{\circ }$). We explain it by the fact that, prior to loading, during their setting on the traction device, the gripping and the flattening of the samples is likely to cause a slight stretch, which induces a traction on the separation lines. However, for samples with an initial azimuth parallel to the traction direction, these lines were most likely sheared or compressed.

The precise microstructural origin of the measured macroscopic polarimetric signal in the myocardium has yet to be identified. However, a very interesting study performed on the skeletal muscle proposes a simplified optical model which can be relevant also to describe the polarimetric effects observed for the myocardium^38^. This model shows that the origin of the measured birefringence can be attributed to the presence of a high density of microscopic fiber structures (myofibrils). Specifically the orientation of these fibers can be visualized using the parameter $\alpha$. Otherwise the measured depolarization $\Delta$ can be produced by the presence of a high density of beads (cellular organelles). By analogy it can be supposed that the birefringence observed on the myocardium is mainly generated by the presence of the cardiomyocytes, the orientation of which can be visualized by using the parameter $\alpha$. In this case the observed depolarization can be mainly attributed to the organelles of the cardiomyocytes. The dense network of collagen fibers composing the extracellular matrix can contribute both to the birefringence and to the depolarization.

**Figure 2:**
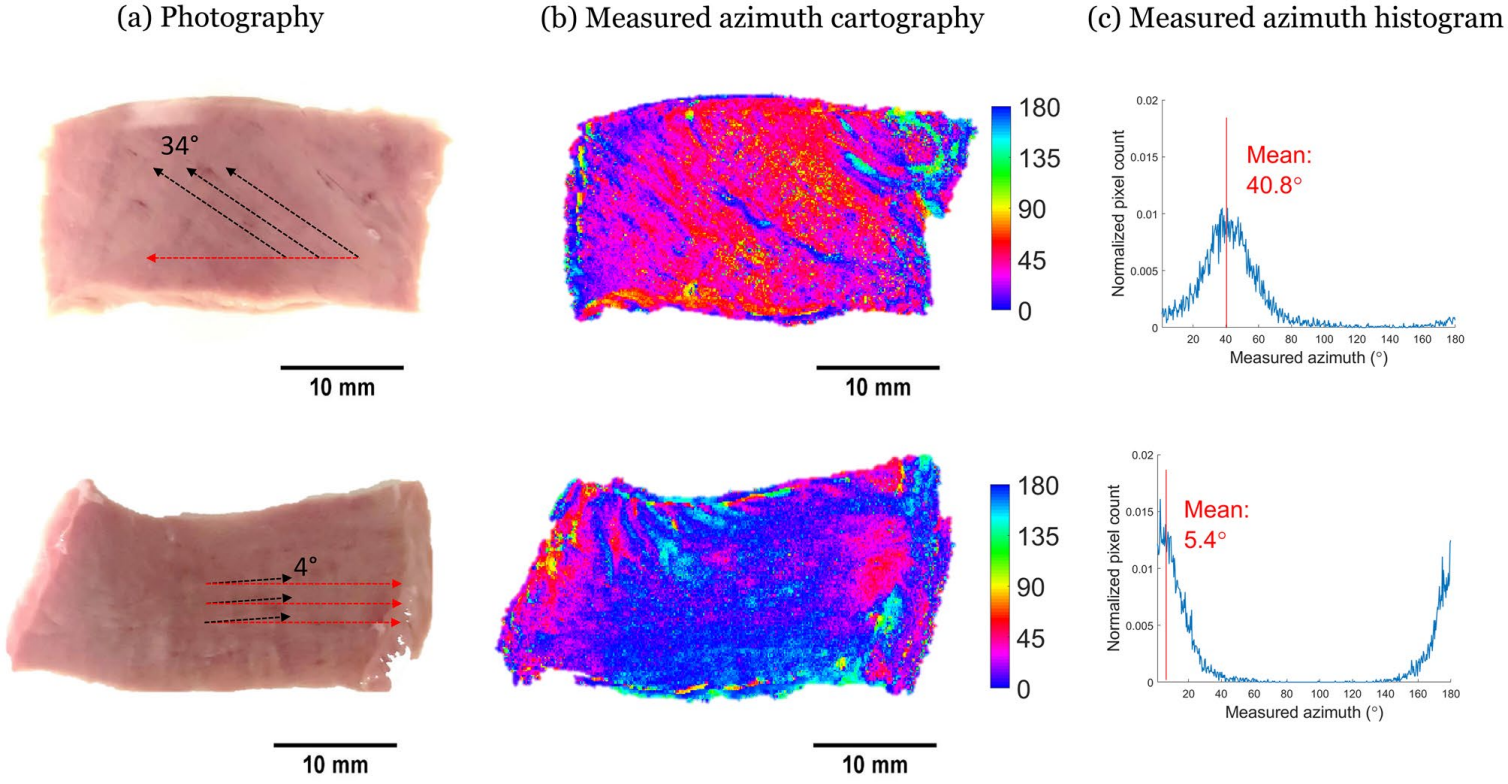
Comparison between (**a**) white-light images and (**b**) azimuth (in $^{\circ }$) maps. (**c**) $\alpha$ histogram of two samples.

We compared classical white-light images and polarimetric images on the same side of the same sample (see Fig. 2). We observed on the white-light images well-ordered mesoscopic lines on the surface of the sample. We manually traced segments over the lines with the best contrast and measured the angle they make with the horizontal orientation using ImageJ. We observed a perfect agreement between this measure and the azimuth measured with the MPI, which confirms that $\alpha$ is a measure of local mesostructure orientation at the surface of the tissue. Our approach doesn’t have the resolution of the azimuth measure and can be biased by the choice of the most contrasted lines. However, this manual measure has already been used in the literature to identify the tissue main orientation^26,28^.

### Stress/stretch behavior

**Figure 3:**
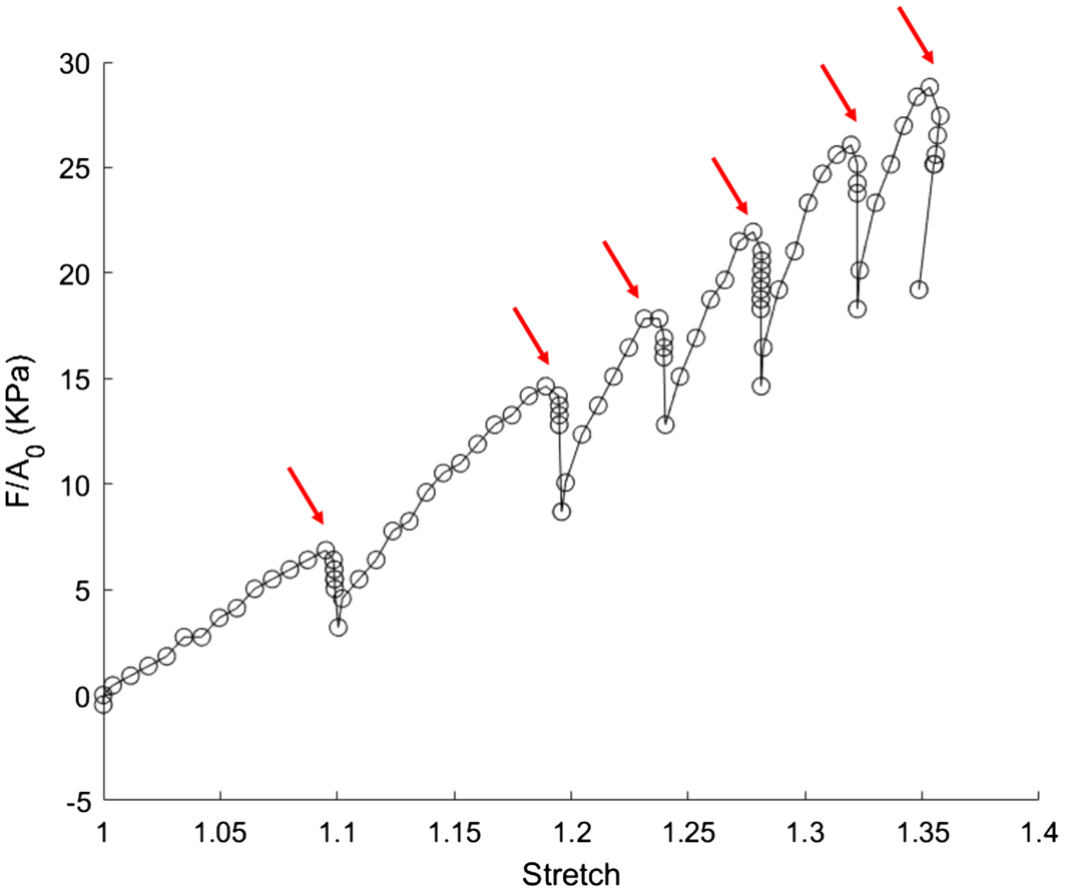
Nominal stress versus optically-measured stretch for sample 8. The relaxations are due to the pauses for MPI acquisition (red arrows).

The macroscopic response of our myocardium samples is similar to what can be found in the literature^22–24^: after observing first a non-linear region, the curve becomes linear (see Fig. 3). An important relaxation occurs when the traction is paused for polarimetric acquisition (around 4 min per acquisition). In order to investigate the effect of the pauses on the global behaviour, we applied continuous stretches on 5 samples, without any pauses, and did not observe any significant differences in the mechanical response.

The heterogeneity of the stretch does not allow it to be simply approximated by the macroscopic one. As polarimetric and deformation measurements are done on two opposite sides of the sample, we investigated if the deformation measured on the lower side can be used to analyse the evolution of the polarimetric measures on the upper side. To do so, a test was done to verify that the stretch measurement gave similar results on both faces. 5 samples were subjected to continuous stretch. Each side was imaged using an optical camera, allowing the computation of a local stretch at a scale of 500 $\upmu$m, using Digital Image Correlation. The deformation fields on both sides were compared. We measured a typical stretch difference around 6% at 40% of applied stretch (see Supplementary Figure 1). As this difference was significantly much smaller than the applied stretch, we concluded that our stretch measure was a good (although non perfect) approximation of the one on the MPI side.

### Polarimetric parameters evolution with traction

**Figure 4:**
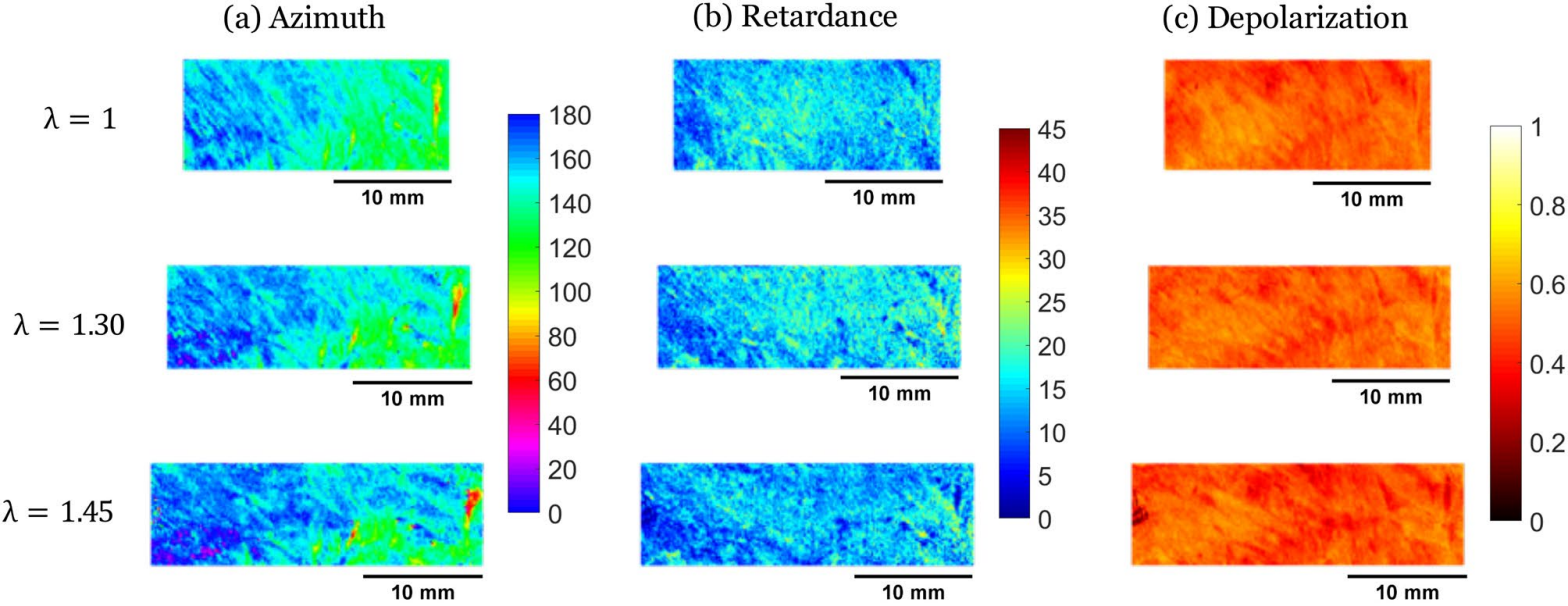
Maps of the Mueller polarimetry parameters [(**a**) Azimuth $\alpha$ (in $^{\circ }$), (**b**) retardance *R* (in $^{\circ }$), (**c**) depolarization $\Delta$] measured on sample 1 at different imposed stretches ($\lambda = 1, \lambda = 1.30,\lambda = 1.45$).

**Figure 5:**
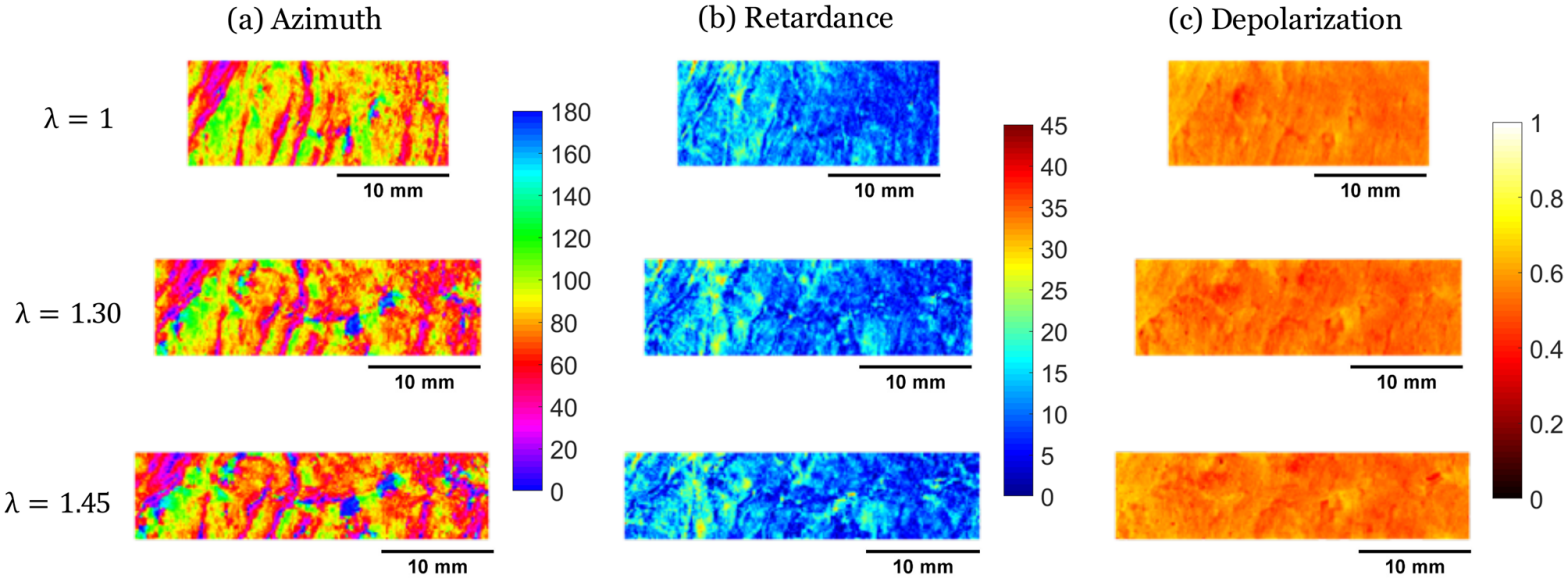
Maps of the Mueller polarimetry parameters [(**a**) Azimuth $\alpha$ (in $^{\circ }$), (b) retardance *R* (in $^{\circ }$), (**c**) depolarization $\Delta$] measured on sample 8 at different imposed stretches ($\lambda = 1, \lambda = 1.30,\lambda = 1.45$).

For each sample, three polarimetric images (of Azimuth $\alpha$, Linear Phase Retardance *R*, and Depolarization $\Delta$) were extracted from the Mueller Matrix, for different levels of stretch. In parallel, DIC was performed on regions of the images generated by the optical camera. To compare the same region of interest, we first registered the polarimetric and the DIC images to superimpose them and match the pixels. Then, we used DIC to deform the initial region, and finally, we cropped both images on the same region all along the traction. The Figs. 4 and 5 show the evolution of the polarimetric parameters at different stretch levels for the same 2 samples as Fig. 1 (see Supplementary Figure 2 for similar figures on all samples).

#### Azimuth

An evolution of the azimuth towards the angle of traction is observed. This is interpreted by the fact that the azimuth measures the microstructure orientation, which naturally tends to align with the traction direction.

**Figure 6:**
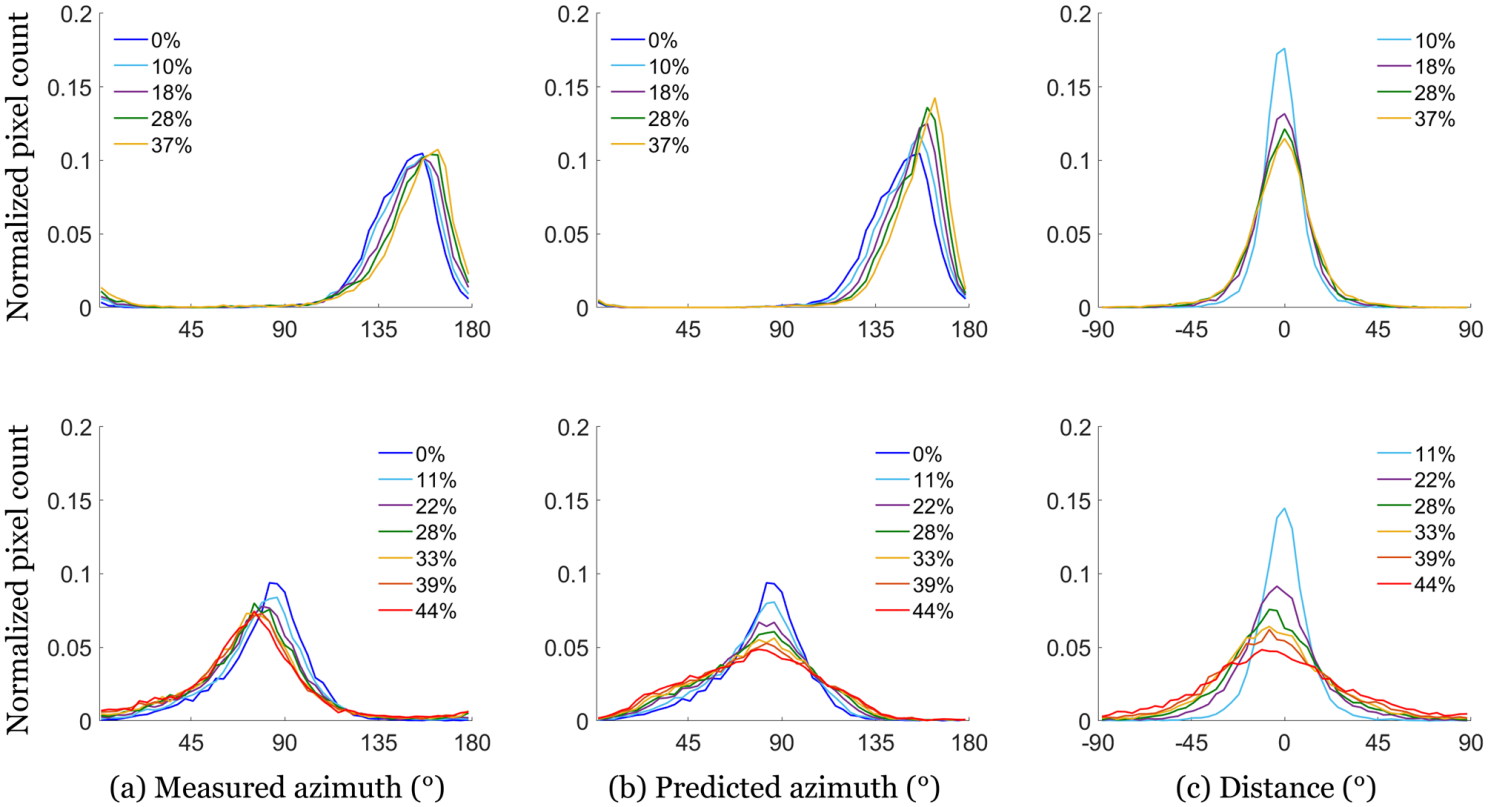
(**a**) Measured azimuth, (**b**) predicted azimuth, and (**c**) angular difference between measured and predicted azimuth distributions for sample 1 ($\langle \alpha \rangle \approx 150^{\circ }$—top) and sample 8 ($\langle \alpha \rangle \approx 80^{\circ }$—bottom), at different stretch levels. The predicted distributions are obtained using the affine assumption.

The simplest way to link the motion of the fibers to the tissue deformation is by adopting the affine assumption, which assumes that the microstructure follows the motion of its surrounding volume^39^. This assumption is widely used in heart multiscale modeling^10,40–42^. It has the main advantage of being purely kinematic. Thus, it allowed us to predict the azimuth evolution, knowing the local stretch and the initial azimuth, and generate maps of the predicted $\alpha$. The difference between the predicted maps and the MPI-measured azimuth maps were then computed. Figure 6a shows the histogram of the measured azimuth at different stretch levels for samples 1 and 8. It also shows the computed histogram at the same stretch levels, using the affine assumption and the initial experimental histogram (Fig. 6b). Finally, the third histogram shows the difference between experimental and computed orientations at each stretch level (Fig. 6c). The results for all samples are available on Supplementary figure 3.

The samples with initial orientations close to the traction direction showed differences between computed and measured azimuth centered on 0 (see Fig. 6 top), indicating that the affine assumption leads to a good approximation of the azimuth evolution. We attribute the dispersion to the fact that we measure the deformation on the opposite side to the azimuth measure.

**Figure 7:**
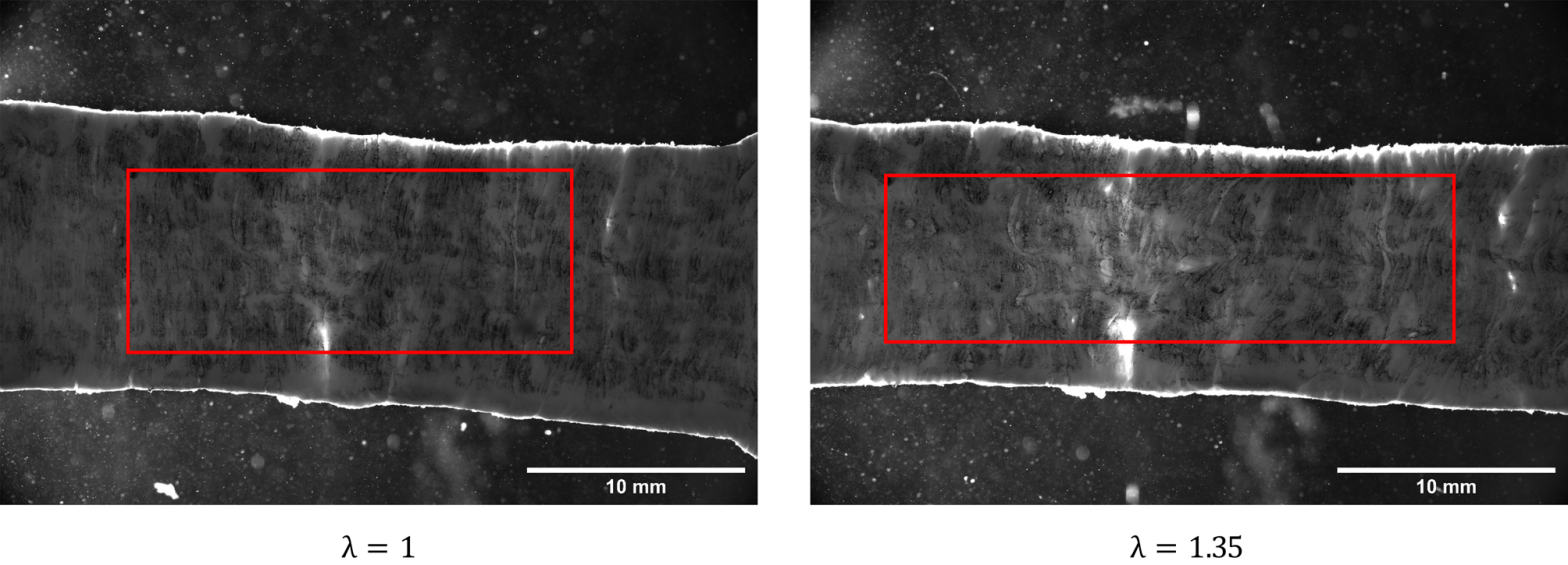
White-light images of the surface used for DIC (opposite to MPI) during the traction of sample 4. Left—prior stretch, $\lambda = 1$, Right—during stretch, $\lambda =1.35$. We observe vertical lines of light grey which open during the traction, that we associate with the separation lines.

However, the samples with an initial azimuth perpendicular to the traction direction ($\langle \alpha \rangle \approx 80^{\circ }$) showed non-centered distributions (see Fig. 6 bottom). The measured azimuth distribution remains almost unchanged during the stretch: as expected, fibers perpendicular to the traction do not rotate with the traction. However, the computed azimuth distribution evolves strongly towards the traction direction, leading to a significant difference between prediction and measure. This can be explained by the existence of separation lines, which open during traction (see Fig. 7), and create discontinuities in the tissue. As the resolution of the stretch measurement is larger than the size of separation line, the measured stretch includes a contribution of both the deformation of the cells and of the lines. When the lines open, they induce an increase in the deformation measure which is not representative of what is experienced by the tissue. This leads to an overestimation of the stretch applied on the cells and thus to errors in the azimuth variations.

#### Retardance

During traction, the retardance does not seem to vary significantly (see Figs. 4, 5, and Supplementary Figure 2) apart in some regions of strong non-affine behavior where it increases slightly with the line opening. It is likely that the imposed deformation is not sufficient to induce a significant modification of the fiber optical environment, but on the separation lines the traction opens gaps between cells, modifying the linear retardance.

#### Depolarization

Depolarization is interpreted as the result of multiple scattering events of the light by small organelles in the cells. During traction, the depolarization changes slightly (see Figs. 4, 5, and Supplementary Figure 2), probably because the organelles are not affected by the deformation. The only significant change is the depolarization decrease on the separation lines when they are perpendicular to the direction of traction. We explain this decrease by the opening of the separation lines, which creates a gap between cardiomyocytes and decreases the tissue density, decreasing the scattering.

## Discussion

In the recent years, the role of the myocardium mesostructure has attracted more and more attention. This is due to the fact that the physiological role of this spatial organization remains unclear^10,11^. However, as the collagen extracellular matrix is strongly modified during heart remodeling (such as infarction^13,17^), it may play a role in the heart remodeling capacities. Developed models in the literature include both the cardiomyocyte and the perimysium layer^20,43^. However, they rely on the affine transformation assumption, which states that local kinematics of each constituent follow the local macroscopic deformation. To our knowledge, this hypothesis has never been experimentally tested, as it requests a simultaneous observation of the macroscopic deformation and the microstructure orientation. In this study, we investigate the question of the cardiomyocyte bundle mechanical response in the myocardial tissue by combining for the first time a traction assay with Mueller polarimetry imaging. These cardiomyocyte bundles group together a few dozen cells, and are around a few hundred of micrometers in thickness. They are often considered as organized in sheetlets^6,7^, although their spatial organization remains undetermined. Not surprisingly, their mechanical properties remain elusive as well^8^.

Mueller Polarimetric Imaging explores the response of the tissue with respect to different polarization states of the illuminating light. Therefore, it probes the tissue organization. Three parameters appear to be of interest in the myocardium: the Azimuth, the Linear Phase Retardance, and the Depolarization. We associate the azimuth $\alpha$ with the cardiomyocyte orientation on the surface (see Fig. 2), and the depolarization $\Delta$ with the scattering events due to organelles and other small disorganized elements in the cardiomyocytes. During traction, azimuth tends to align with the traction direction, indicating that the fibers align with traction (see Fig. 6), for all samples except for those with an initial azimuth perpendicularly oriented to the traction. The motion of the fibers is well described by the affine assumption, which states that the fibers follow the deformation of the tissue they are embedded in. This emphasizes the cohesion of the cardiomyocytes together. The depolarization, as well as the retardance, do not appear to be modified by the traction. This indicates that the imposed deformations do not affect the optical anisotropy and thus are not sufficient to deform the cardiomyocytes at a perceptible level.

We observe “separation lines” in which the azimuth varies a lot on few micrometers, and in which the depolarization is smaller (see Fig. 1). These lines are initially separated by few hundreds of micrometers. We hypothesize that these lines are the perimysium, the collagen layer surrounding the border of the cell aggregates. They have the adequate size, and show a sudden change in the orientation. The lower depolarization indicates that the tissue is less dense or contains less diffusive elements. During traction, we observe that these lines become larger for samples with azimuth perpendicular to the traction direction. At the same time, the depolarization decreases in the line. Therefore, these lines appear to open under traction as observed on samples with azimuth near $90^{\circ }$, as well as on optical images (see Fig. 7). Samples with other orientations do not exhibit an opening of these separation lines, nor do they show a non-affine response. This supports that the perimysium is resistant against shear and compression, but weak against traction, as we may expect for a thin loosely-connected layer of collagen.

To confirm the mechanical role of these separation lines, it will be of interest to advance more complex mechanical loadings after having confirmed that they are really the borders of sheetlet. An improvement of the resolution of the Mueller polarimetric set-up will enable to achieve a more precise view of the microstructure. But most importantly, further developments will be strongly helped by an optical model of the signal including the cardiac microstructure, so that we can associate more precisely each element to a polarimetric parameter, as well as the size of probed volume.

The obtained results prove the feasibility to measure the mechanical properties of the myocardium mesostructure. These experimental measures can be used as inputs for the mathematical models to determine myocardial mechanical properties, although our results show that affine-based models are unlikely to reproduce correctly our observations. With such approach one can compare healthy and remodeled or pathological myocardium samples, and see how the mechanical response is modified at a mesoscale—a scale not investigated so far. However direct clinical applications will request further studies.

## Methods

### Sample preparation

**Figure 8:**
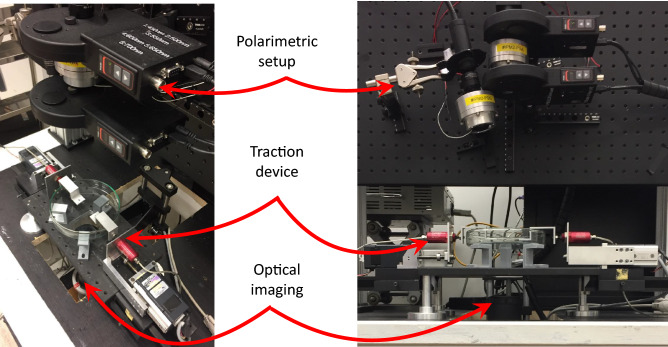
Global and close views of our experimental setup.

In total, 9 samples extracted from porcine left ventricles were studied. The hearts were purchased at the local butcher, stored in a cooling box during transportation, and tested the same day. The left ventricle was first isolated. Then, using a slicing machine, the pericardium and the myocardium were separated, and thin slices of myocardial tissue were cut (average thickness $2.4 \pm 0.5$ mm). From these slices, strips of $30\times 10$ mm were cut with a scalpel. The dominant orientation of the microstructure on the upper surface of each slice was visually identified with respect to the strip length. To measure the displacement field, a speckle pattern was created on one of the surfaces of each sample by tapping a sponge sunk in Indian ink. The freshly cut samples were then mounted on a custom-made uniaxial traction machine^36^ (see Fig. 8). The sample was then immersed in a solution of phosphate-buffered saline (PBS). In our setup, the upper surface of the sample was observed by MPI, while the lower surface (the stained one) was observed by a standard optical camera to measure the stretch field. Once the sample in place, its dimensions were measured using a digital caliper.

### Mueller polarization

A custom-made sequential multispectral Mueller polarimetric imager (MPI) in backscattering configuration, designed and constructed at LPICM, was used to analyze myocardial samples. It is an upgraded version of the one described in^32^. Briefly, a Polarization State Generator (PSG) temporally modulates the polarization of the light impinging on the sample to be explored by consecutively generating four independent probing polarization states which are described by four Stokes vectors and regrouped together as the columns of a modulation matrix called $\underline{\underline{W}}$. The light backscattered by the sample then passes through a Polarization State Analyzer (PSA) placed in front of the detector. Each of the four polarization states produced by the PSG, after interacting with the sample, is analyzed trough four independent consecutive configurations of the PSA which are described by four Stokes vectors and grouped together as the rows of an analysis matrix called $\underline{\underline{A}}$. Described procedure enables to acquire 16 intensity images which are stacked in a 4x4 matrix $\underline{\underline{B}}$ given by:

1 $$\begin{aligned} \underline{\underline{B}}=\underline{\underline{A}}.\underline{\underline{M}}.\underline{\underline{W}}, \end{aligned}$$

where $\underline{\underline{M}}$ is the unknown Mueller matrix of the analyzed sample. If $\underline{\underline{W}}$ and $\underline{\underline{A}}$ have been previously accurately determined through a calibration process, the Mueller matrix $\underline{\underline{M}}$ of the sample can be obtained from Eq. (1).

The Eigenvalue Calibration Method, widely described in^44^, has been used here to calibrate the system. The PSG and PSA have been constructed by using ferroelectric liquid crystals as explained in^45^. The light is delivered by a 150 Watt halogen source (Olympus CLH-CS, USA). and guided to the MPI by means of a liquid light guide (Thorlabs LLG0538-6, USA). Appropriate collimation optics was placed at the outlet of the liquid guide to produce a bright spot 6 cm in diameter at a working distance of about 30 cm corresponding to the sample position. A monochromatic CCD-camera (f080b Allied Vision, Germany, 512x386 pixels in binning mode) has been used as detector. It is provided with an electronics enabling to acquire 16-bit intensity images. The CCD-camera is provided of an objective of focal length f = 25mm and it has been positioned at a distance of about 20 cm from the sample in order to obtain a field of view of about $5\times 4\,\text{ cm}^2$, corresponding to a nominal resolution of about 100 $\upmu$m/pixel. A wheel containing several dichroic filters is placed in front of the CCD-camera, enabling to perform measurements between 450 and 700 nm with steps of 50 nm and a bandwidth of 40 nm for each selected wavelength. In particular, the wavelength at 550 nm has been selected for the experiments described in this study.

The Mueller matrix $\underline{\underline{M}}$ of a sample enables its comprehensive polarimetric characterization. Once the Mueller matrix has been measured, the sample’s polarimetric properties can be retrieved by a number of algebraic procedures. In this study, we made the choice to interpret these images by applying the polar decomposition proposed by Lu and Chipman^37^. This decomposition enables to describe a Mueller matrix M as the product of three matrices:

2 $$\begin{aligned} \underline{\underline{M}}=\underline{\underline{M}}_\Delta .\underline{\underline{M}}_R.\underline{\underline{M}}_D, \end{aligned}$$

where $\underline{\underline{M}}_\Delta$ is the Mueller matrix of a depolarizer, $\underline{\underline{M}}_R$ of a birefringent medium and $\underline{\underline{M}}_D$ of a diattenuator. In particular, the main polarimetric properties observed on myocardial tissue are the depolarization and the linear birefringence. This last can be quantified by two parameters which are the phase retardance and the orientation of its fast (or equivalently the slow) axis also named azimuth. Depolarization and linear phase retardance are linked to scattering and anisotropy of a tissue on a microscopic scale. The depolarization is expressed as:

3 $$\begin{aligned} \Delta = 1-\frac{\vert a\vert + \vert b\vert + \vert c\vert }{3}, \end{aligned}$$

where *a*, *b* and *c* are the eigenvalues of $\underline{\underline{M}}_\Delta$. $\Delta$ reflects the total depolarization power of the Mueller matrix, and includes the sample ability to depolarize both linear and circular polarization states. It ranges from 0 in the case of non-depolarizing to 1 for a pure depolarizing sample.

The phase retardance can be obtained by:

4 $$\begin{aligned} R = \arccos \left( {\frac{tr\left( {\underline{\underline{M}}_R}\right) }{2}-1}\right) . \end{aligned}$$

It is measured in degrees and for biological tissues generally spans in the full range between 0 and $180^{\circ }$.

Finally the azimuth is given by:

5 $$\begin{aligned} \alpha = \frac{1}{2}\arctan \left( {\frac{\underline{\underline{M}}_{R24}}{\underline{\underline{M}}_{R43}}}\right) . \end{aligned}$$

With this formula, the azimuth varies in the range between 0 and $\,90^{\circ }$. To avoid recurring jumps and to facilitate the reading on macroscopic maps, we extended this range so that $\alpha$ spans between 0 and $\,180^{\circ }$^33^.

### Mechanical assay

The mechanical traction was done using a custom-made traction device made of two symmetric motors^36^, equipped with load cells (LPM200, 2lb, Futek, USA), on which the sample was attached (see Fig. 8). After immersion in PBS, we slowly stretched the sample to flatten it, while keeping the force below the noise level. Once the sample was flat (seen with the naked eye), its size was measured using a digital caliper. Then a traction at 0.1 mm/s (around 0.3%.s$^{-1}$) was exerted. The force was recorded every second. At every 10% of the initial sample length, the motors were stopped for the acquisition of the polarimetric images.

In parallel, high definition images of the lower side of the sample were taken (0.33 fps), using a 8-bit CCD camera (Allied Vision GX6600, 6576 (H) $\times$ 4384 (V), Allied Vision, Germany) connected to a telecentric lens (Opto Engineering TC16M036, Opto Engineering, Italy). The camera has a field of view of $4384\times 6576$ px, each pixel corresponding to a physical square of $5.5 \times 5.5\,\upmu$m. The lighting (and thus the optical acquisition) was interrupted during the polarimetric acquisition.

Using these images, local-based Digital Image Correlation (DIC) was performed (CorrelManuV 1.66, Michel Bornert^46^) to determine the displacement and stretch fields. The correlation was performed on positions separated by 100 px (500 $\upmu$m), with local domains of 100 px. Thus, the displacement is measured on a grid of square pixels of a size of 100 px (550 $\upmu$m in physical unit). The correlation was performed on the region of the sample which remained in the camera field of view during the whole traction (typically $30\times 15$ displacement points).

### Deformation on the upper surface

As the polarimetric and deformation measurements are done on two different surfaces of the sample, we also checked if our stretch measurement gave similar results on both sides. To do so, the polarimetric set-up was replaced by an optical camera (Allied Vision GX3300, 3296 (H) $\times$ 2472 (V), Allied Vision, Germany). 5 samples, extracted from porcine left ventricles and of dimensions approximately $30\times 10\times 2.5\,\hbox {mm}^3$, were tested as previously described, without the pauses for the polarimetric acquisition.

### Images superposition

In order to compare mechanical and polarimetric data, the information obtained on the two sides had to be extrapolated on the same location. To do so, we first registered the two images so that both were aligned in the same coordinate system. The registration was done using a “feature-based” technique, consisting in finding a few matching features on both images, and then computing a transformation matrix $\underline{\underline{N}}$ that matches points $\underline{X}$ from the first image and their corresponding points $\underline{X}'$ in the second image by a least-square minimization. The feature detection was done manually using Fiji, and the minimization was done in Matlab. The maximum residual obtained using the Matlab minimization was of 20 pixels, which represents a 4% error. The transformation matrix $\underline{\underline{N}}$ concatenates a translation $\underline{\underline{T}}$, a scaling ($\underline{\underline{S}}$), and a rotation of the plane $\underline{\underline{R}}$ such that:

6 $$\begin{aligned} \underline{\underline{N}} = \underline{\underline{T}} \cdot \underline{\underline{R}} \cdot \underline{\underline{S}}. \end{aligned}$$

The scaling avoids the need to convert the pixel size into physical length: we preferred to work in pixel of the upper camera. The two images were cropped to observe only the same region of interest.

Once the images were registered, we determined the displacement $\underline{u}(\underline{X})$, and the associated deformation gradient $\underline{\underline{F}}(\underline{X})$ for each position $X_i$ at which the polarimetric parameters were measured. As the DIC image has a lower resolution (10x larger) than the polarimetric image, we used a bilinear extrapolation between the nearest displacement measures to determine the deformation $\underline{\underline{F}}(\underline{X_j})$ in between.

Note that the positions $X_j$ were in the initial polarimetric image (prior to loading), as the displacement field was defined in the sample reference configuration. Using the measured displacement, it was possible to determine the values of the mechanical parameters at each position $x_j$ in the subsequent polarimetric image.

## Supplementary information

Supplementary Figures.
