# Supplementary material for: Microstructural deformation observed by Mueller polarimetry during traction assay on myocardium samples
Source: Sci Rep. 2020 Nov 25;10:20531. doi: 10.1038/s41598-020-76820-w (PMC7688642; doi:10.1038/s41598-020-76820-w)
Supplement: Supplementary file 1 — Supplementary Figures. [file 41598_2020_76820_MOESM1_ESM.pdf]

Supporting Information

Microstructural deformation observed by Mueller Polarimetry during traction assay on myocardium samples

Nicole Tueni[1,2,+], *Jérémy Vizet*[3,+], *Martin Genet*[1,2], *Angelo Pierangelo*[3], *Jean-Marc Allain*[1,2,*]

*Corresponding Author: angelo.pierangelo@polytechnique.edu ;

jean-marc.allain@polytechnique.edu

+These authors contributed equally to this work.

[1] LMS, CNRS, École Polytechnique, Institut Polytechnique de Paris, Palaiseau, France

[2] Inria, Palaiseau, France

[3] LPICM, CNRS, École Polytechnique, Institut Polytechnique de Paris, Palaiseau, France

<u>Supplementary figure 1:</u>

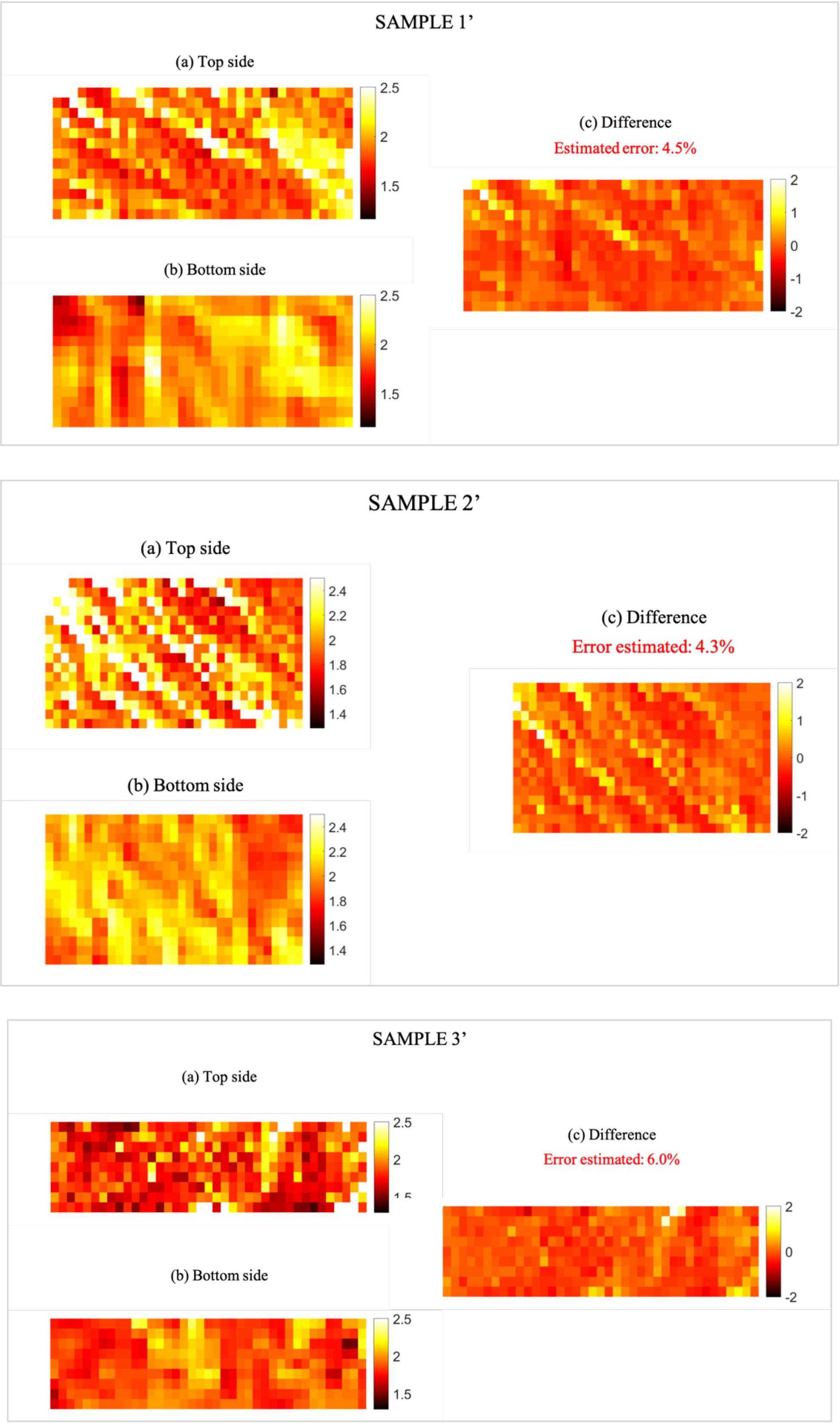

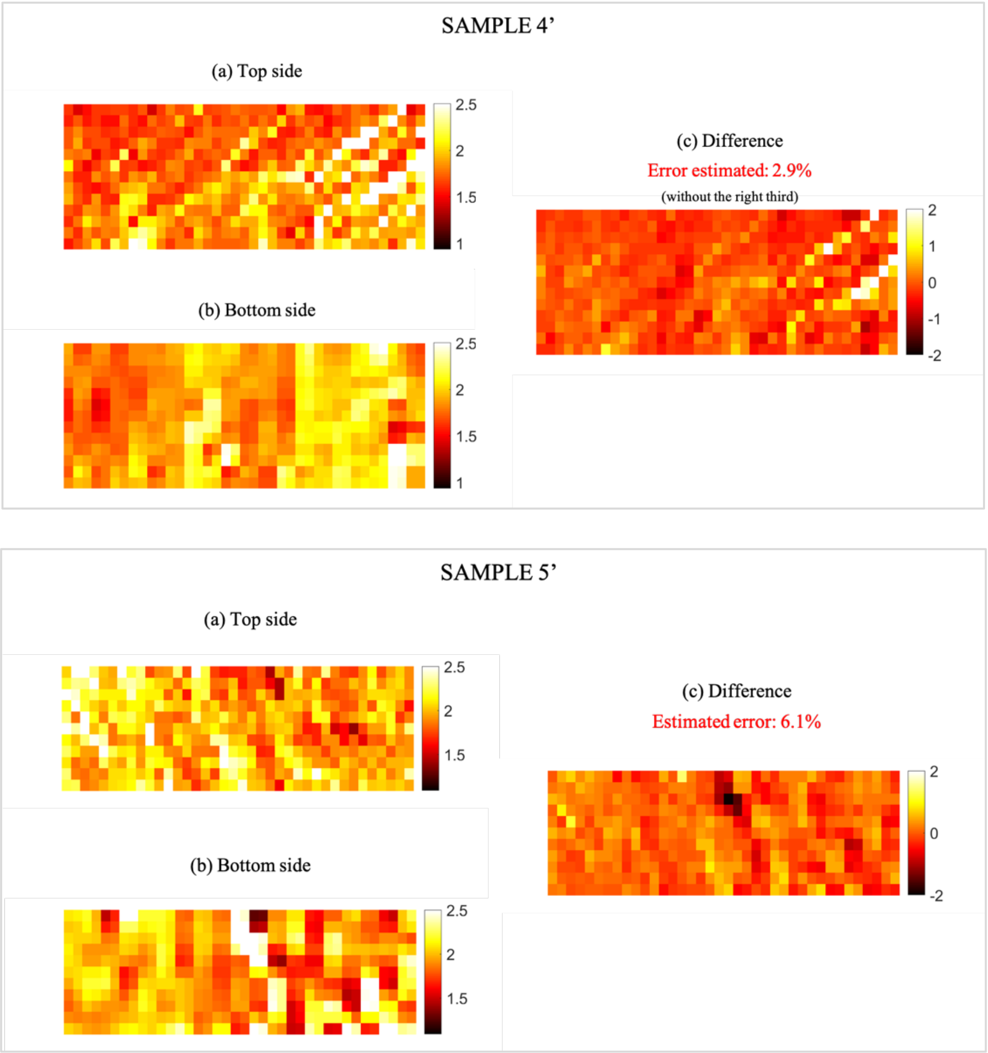

Figure S1. Comparison of the stretch measured by DIC on both sides of the same sample. (a) top and (b) bottom measures of each sample. (c) Difference $\boldsymbol{C_{top}} - \boldsymbol{C_{bottom}}$ of the two measures, as well as the estimation of error on the stretch based on the standard deviation of this difference.

Supplementary figure 2:

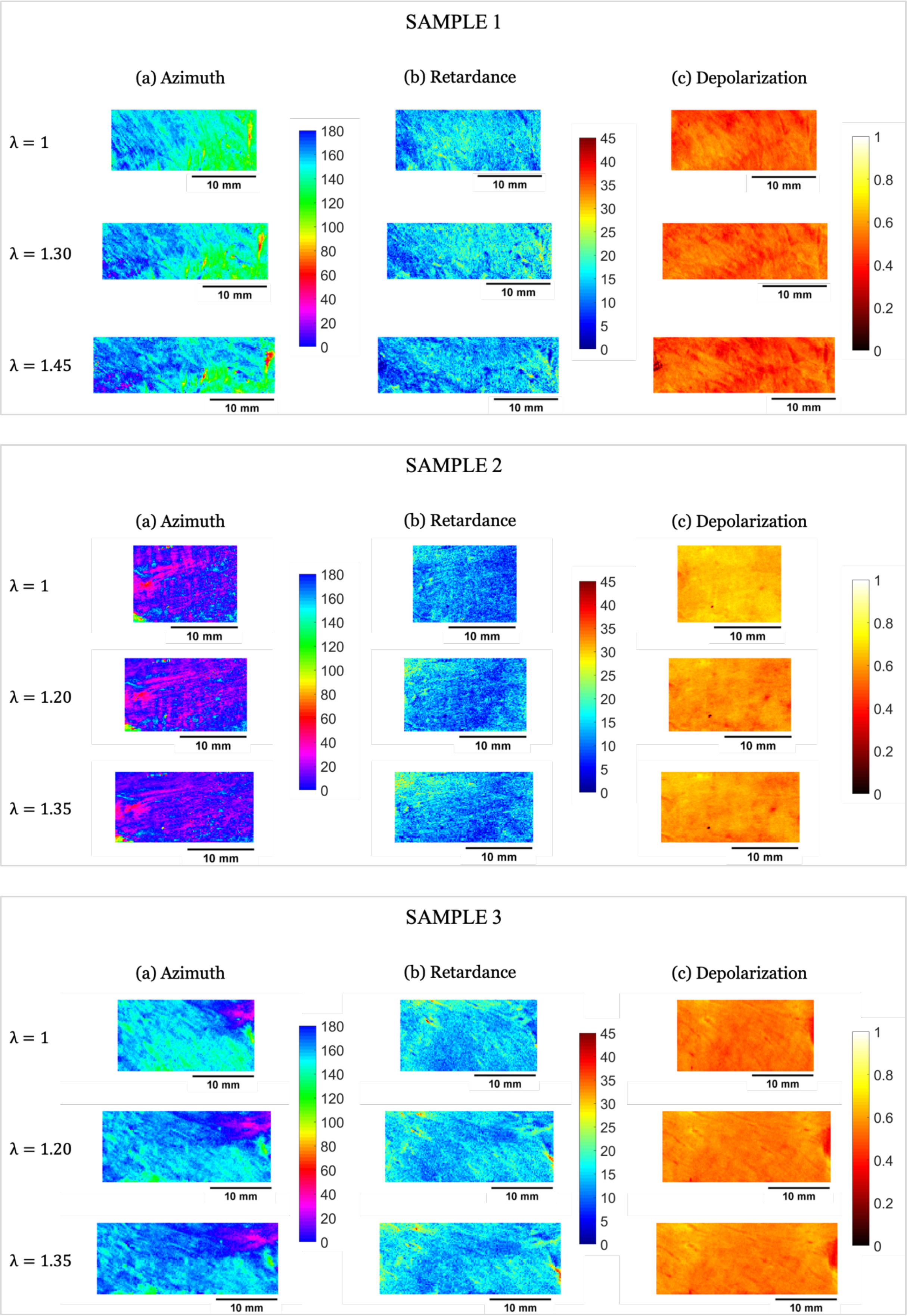

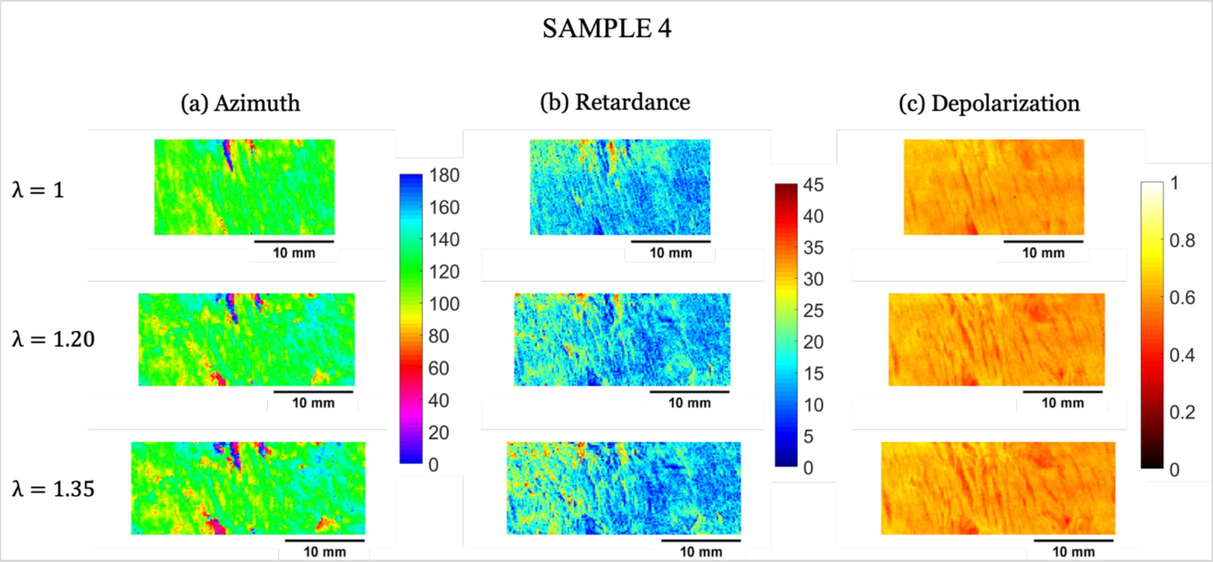
SAMPLE 4
(a) Azimuth
(b) Retardance
(c) Depolarization
λ = 1
λ = 1.20
λ = 1.35
10 mm
180 160 140 120 100 80 60 40 20 0
45 40 35 30 25 20 15 10 5 0
1 0.8 0.6 0.4 0.2 0

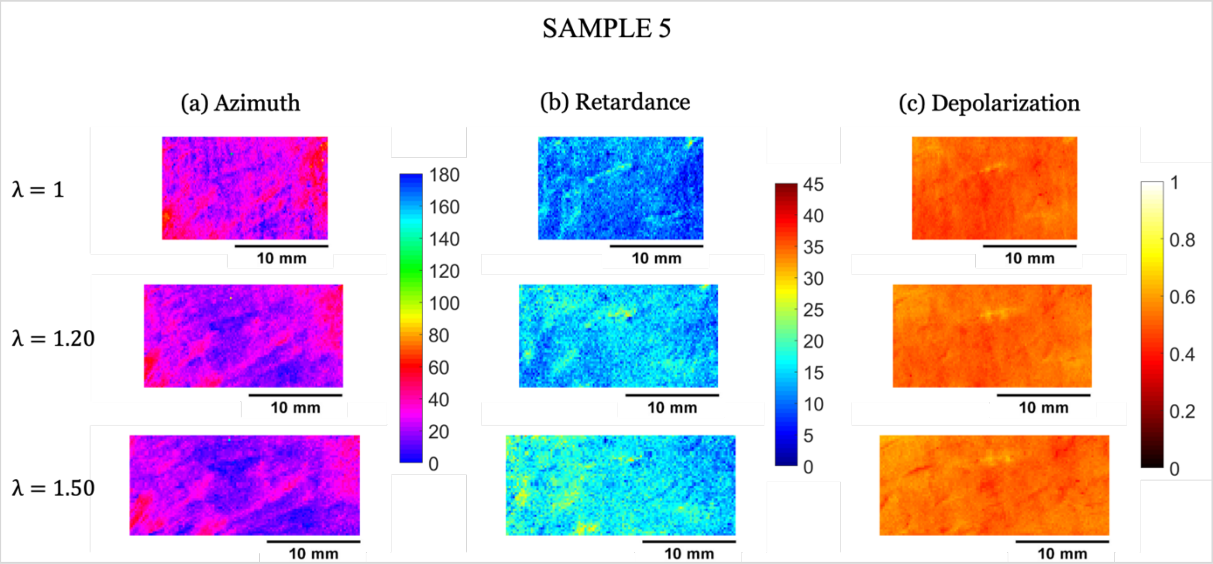
SAMPLE 5
(a) Azimuth
(b) Retardance
(c) Depolarization
λ = 1
λ = 1.20
λ = 1.50
10 mm
180 160 140 120 100 80 60 40 20 0
45 40 35 30 25 20 15 10 5 0
1 0.8 0.6 0.4 0.2 0

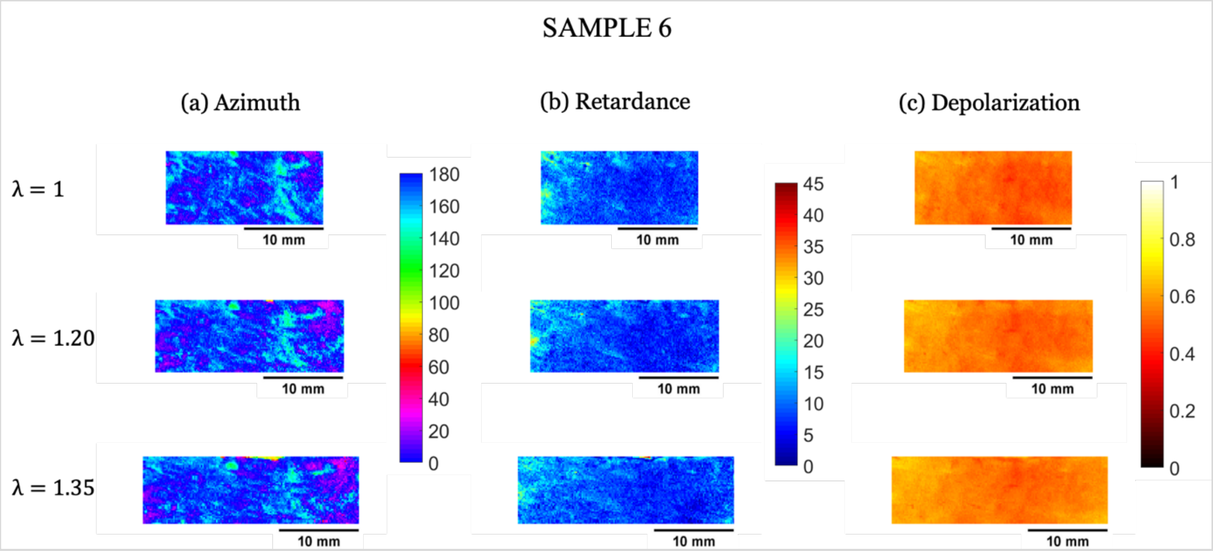
SAMPLE 6
(a) Azimuth
(b) Retardance
(c) Depolarization
λ = 1
λ = 1.20
λ = 1.35
10 mm
180 160 140 120 100 80 60 40 20 0
45 40 35 30 25 20 15 10 5 0
1 0.8 0.6 0.4 0.2 0

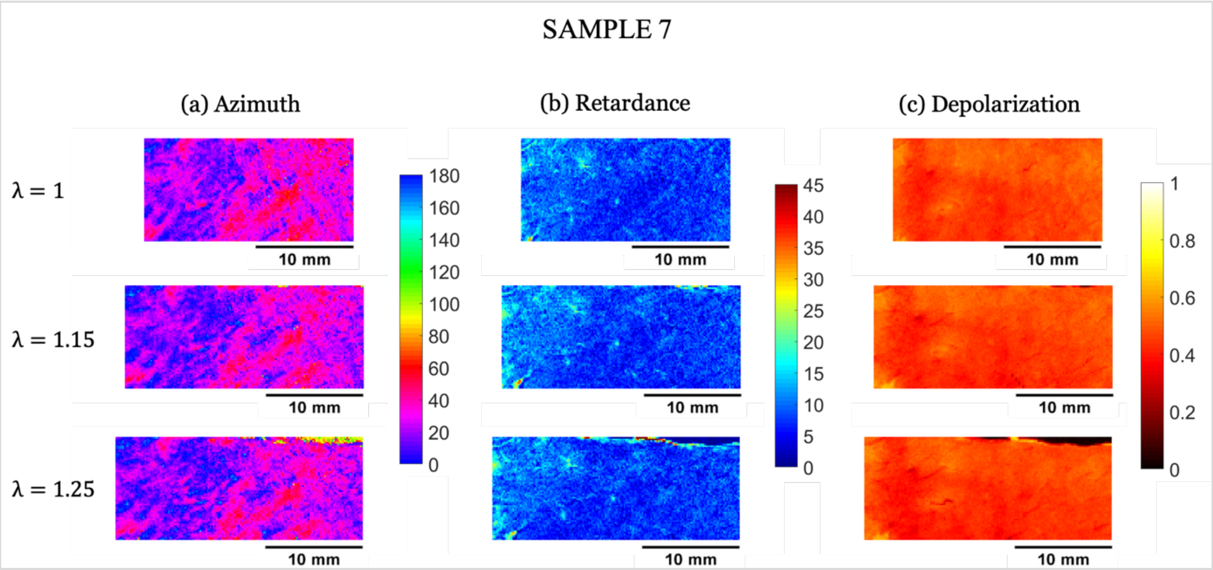
SAMPLE 7
(a) Azimuth
(b) Retardance
(c) Depolarization
λ = 1
λ = 1.15
λ = 1.25
10 mm
180 160 140 120 100 80 60 40 20 0
45 40 35 30 25 20 15 10 5 0
1 0.8 0.6 0.4 0.2 0

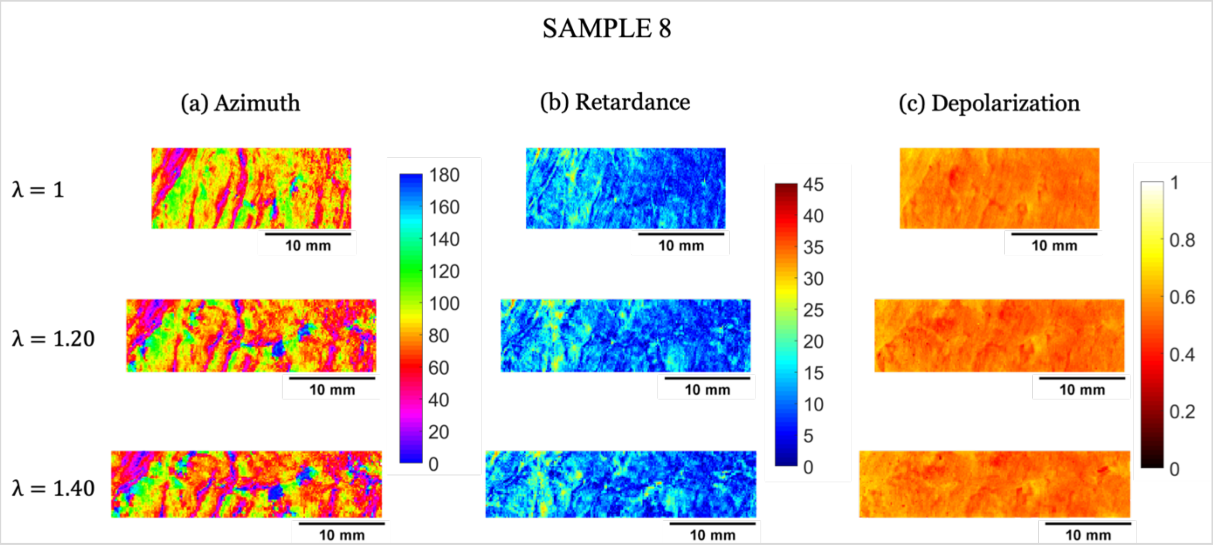
SAMPLE 8
(a) Azimuth
(b) Retardance
(c) Depolarization
λ = 1
λ = 1.20
λ = 1.40
10 mm
180 160 140 120 100 80 60 40 20 0
45 40 35 30 25 20 15 10 5 0
1 0.8 0.6 0.4 0.2 0

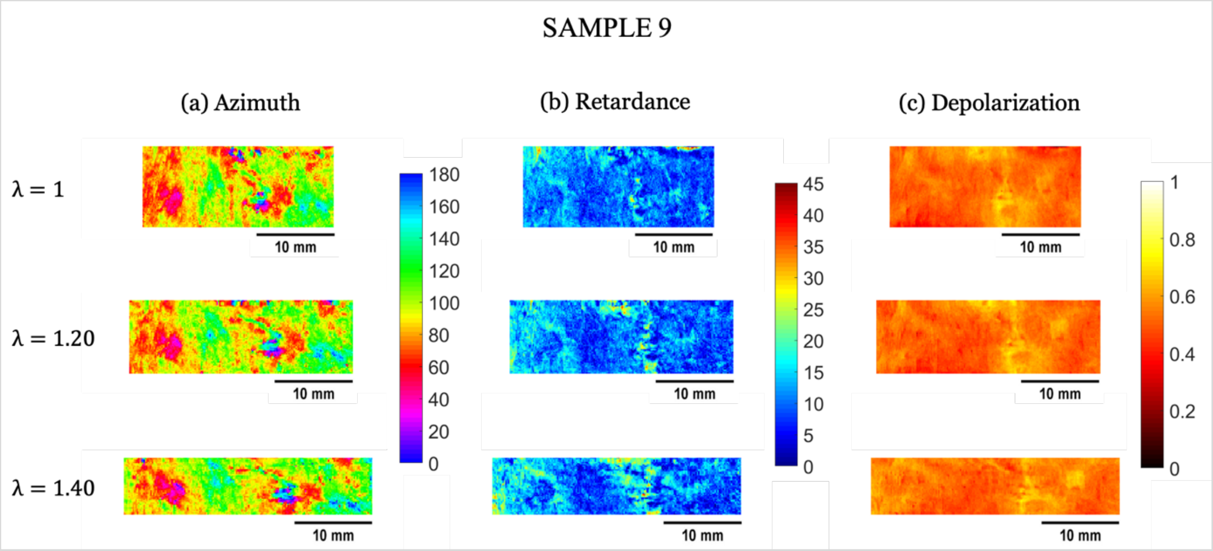
SAMPLE 9
(a) Azimuth
(b) Retardance
(c) Depolarization
λ = 1
λ = 1.20
λ = 1.40
10 mm
180 160 140 120 100 80 60 40 20 0
45 40 35 30 25 20 15 10 5 0
1 0.8 0.6 0.4 0.2 0

Figure S2. Maps of the Mueller polarimetry parameters (a - Azimuth (in °), b - Retardance (in °), c - Depolarization) measured at the different imposed stretches (indicated on the left), for all the samples.

Supplementary figure 3:

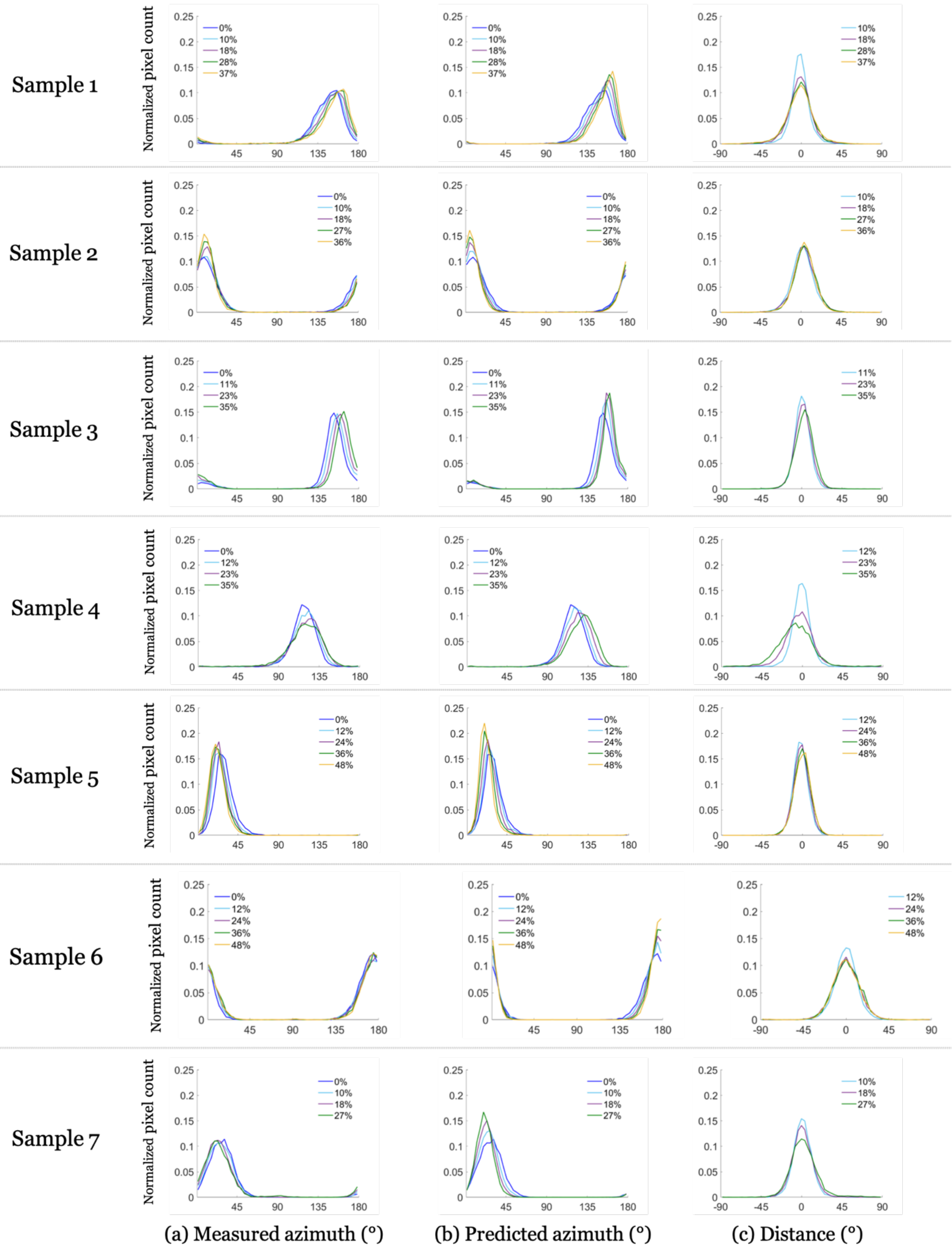

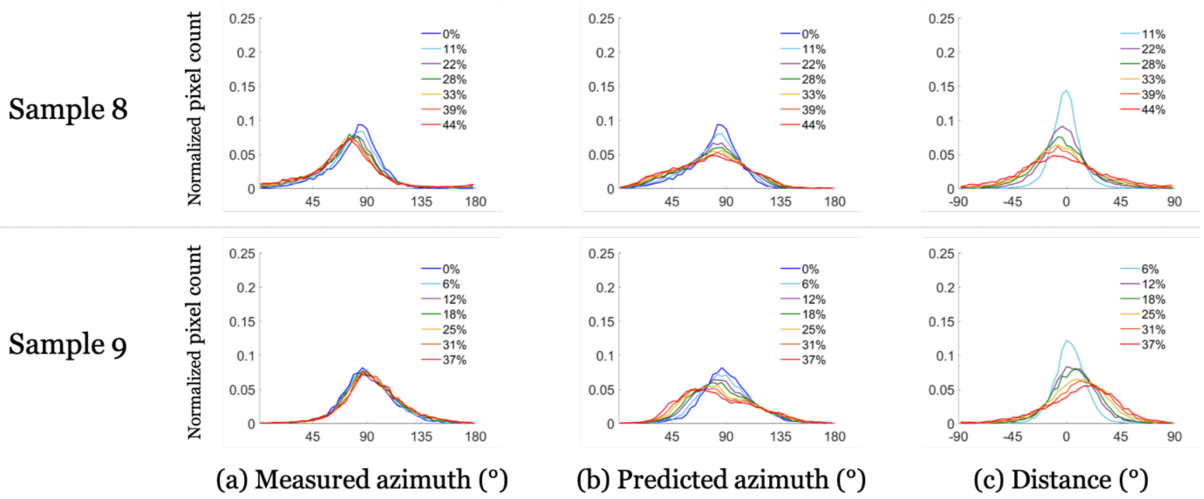

Figure S3. Histogram of the polarimetric parameters at different stretch level for each sample. a - Measured azimuth, b - Predicted azimuth, and c - Angular difference between measured and predicted azimuth distributions for each sample. The predicted distributions are obtained using the affine assumption.
